# A Novel Serum tsRNA for Diagnosis and Prediction of Nephritis in SLE

**DOI:** 10.3389/fimmu.2021.735105

**Published:** 2021-11-11

**Authors:** Ping Yang, Xiaoshan Zhang, Shanshan Chen, Yue Tao, Mingzhe Ning, Yijia Zhu, Jun Liang, Wei Kong, Bo Shi, Zhiyang Li, Han Shen, Yanbo Wang

**Affiliations:** ^1^ Department of Clinical Laboratory, The Affiliated Drum Tower Hospital of Nanjing University Medical School, Nanjing, China; ^2^ College of Life Science, Yangtze University, Jingzhou, China; ^3^ Department of Rheumatic Immunology, The Affiliated Drum Tower Hospital of Nanjing University Medical School, Nanjing, China; ^4^ Department of Clinical Laboratory, Nanjing Jiangning District Hospital of Traditional Chinese Medicine (TCM), Nanjing, China; ^5^ Nanjing Drum Tower Hospital Center of Molecular Diagnostic and Therapy, State Key Laboratory of Pharmaceutical Biotechnology and Department of Physiology, Jiangsu Engineering Research Center for MicroRNA Biology and Biotechnology, NJU Advanced Institute of Life Sciences (NAILS), School of Life Sciences, Nanjing University, Nanjing, China

**Keywords:** tsRNA, SLE, LN, biomarker, ncRNA (non coding RNA), diagnosis

## Abstract

**Objective:**

Dysregulation of transfer RNA (tRNA)-derived small noncoding RNA (tsRNA) signatures in human serum has been found in various diseases. Here, we determine whether the signatures of tsRNAs in serum can serve as biomarkers for diagnosis or prognosis of systemic lupus erythematosus (SLE).

**Methods:**

Initially, small RNA sequencing was employed for the screening serum tsRNAs obtained from SLE patients, followed by validation with TaqMan probe-based quantitative reverse transcription-PCR (RT-PCR) assay. Receiver operating characteristic (ROC) curve analysis was used to assess the diagnostic efficacy. The biological functions of tsRNAs were identified by Gene Ontology (GO) and Kyoto Encyclopedia of Genes and Genomes (KEGG) assay.

**Results:**

We first analyzed tsRNA signatures in SLE serum and identified that tRF-His-GTG-1 was significantly upregulated in SLE serum. The combination of tRF-His-GTG-1 and anti-dsDNA could serve as biomarkers for diagnosing SLE with a high area under the curve (AUC) of 0.95 (95% CI = 0.92–0.99), sensitivity (83.72%), and specificity (94.19%). Importantly, the noninvasive serum tRF-His-GTG-1 could also be used to distinguish SLE with LN or SLE without LN with AUC of 0.81 (95% CI, 0.73–0.88) and performance (sensitivity 66.27%, specificity 96.15%). Moreover, the serum tsRNA is mainly secreted *via* exosome and can directly target signaling molecules that play crucial roles in regulating the immune system.

**Conclusion:**

In this study, it has been demonstrated for the first time that serum tsRNAs can be employed as noninvasive biomarkers for the efficient diagnosis and prediction of nephritis in SLE.

## Introduction

Systemic lupus erythematosus (SLE) is a representative autoimmune disease leading to systemic autoimmunity resulting in various organ damage. SLE mainly occurs in women of childbearing age characterized with volcanic autoantibodies (1–4). Both genetic and environmental factors are considered to be involved, but the precise pathogenesis of SLE is still unclear. The current diagnosis of SLE relies mainly on the clinical manifestations and laboratory tests, including antinuclear antibody (ANA), anti-dsDNA, and anti-Smith antigen (anti-Sm) (5, 6). However, due to the diversity of presentation, the early diagnosis of SLE remains a challenge.

The kidney, a vital organ in the human body, is severely affected by SLE (7). Lupus nephritis (LN) is one of the most severe organ manifestations of SLE and a kind of glomerulonephritis. There are six different histological classes of LN differentiated by their distinct manifestations and severities of renal involvement in SLE (8). LN appears in most SLE patients within 5 years of diagnosis. LN hugely contributes to the severity of the SLE, as 10% of LN patients acquire end-stage renal disease (ESRD) (7). Clearly, early diagnosis of LN and timely initiation of treatment are critical to prevent disease progression. At present, 24-h urine protein quantification and kidney biopsy are the most used methods for LN diagnosis clinically. However, these methods have many drawbacks, such as inaccurate timing, partial urine sample loss during urine retention, poor patient compliance for urine protein test, and invasiveness of the procedure for kidney biopsy. Therefore, it is urgent to explore new biomarkers to distinguish LN from SLE.

Small noncoding RNAs are a group of RNA molecules that are shorter than 200 nucleotides (nt) without coding potential, such as microRNA (miRNA), transfer RNA (tRNA)-derived small noncoding RNA (tsRNA), and PIWI-interacting RNAs (piRNAs) (9). Among them, tsRNAs can spring up from mature tRNA or tRNA precursors and cleaved by enzymes such as Dicer and angiogenin (10, 11). Several reports indicated that tsRNAs play vital roles in the pathological and physiological processes, including cancer (12–16), neurodegenerative disease (17, 18), and metabolic disease (19, 20). However, no study has been done yet to test serum tsRNAs in the context of autoimmune diseases.

Recent studies from our group (21, 22) and others (23, 24) have found that circulating nucleic acids (such as miRNAs) in body fluids can serve as potential biomarkers for detecting various diseases, including immune diseases. It has been established that tsRNAs are stable in circulation (25). Furthermore, recent research has shown that serum tsRNAs are present in high proportion (~70%) than miRNAs (26). However, the diagnostic usefulness and roles of serum tsRNAs, particularly for SLE, remain unclear.

In this study, we used RNA sequencing, qRT-PCR validation, and ROC curve analysis, leading to identifying SLE-associated tsRNA signatures in human serum. The serum tsRNA profile showed significant potential as a noninvasive biomarker for diagnosing and predicting LN in SLE.

## Methods

### Experimental Design

**Figure 1:**
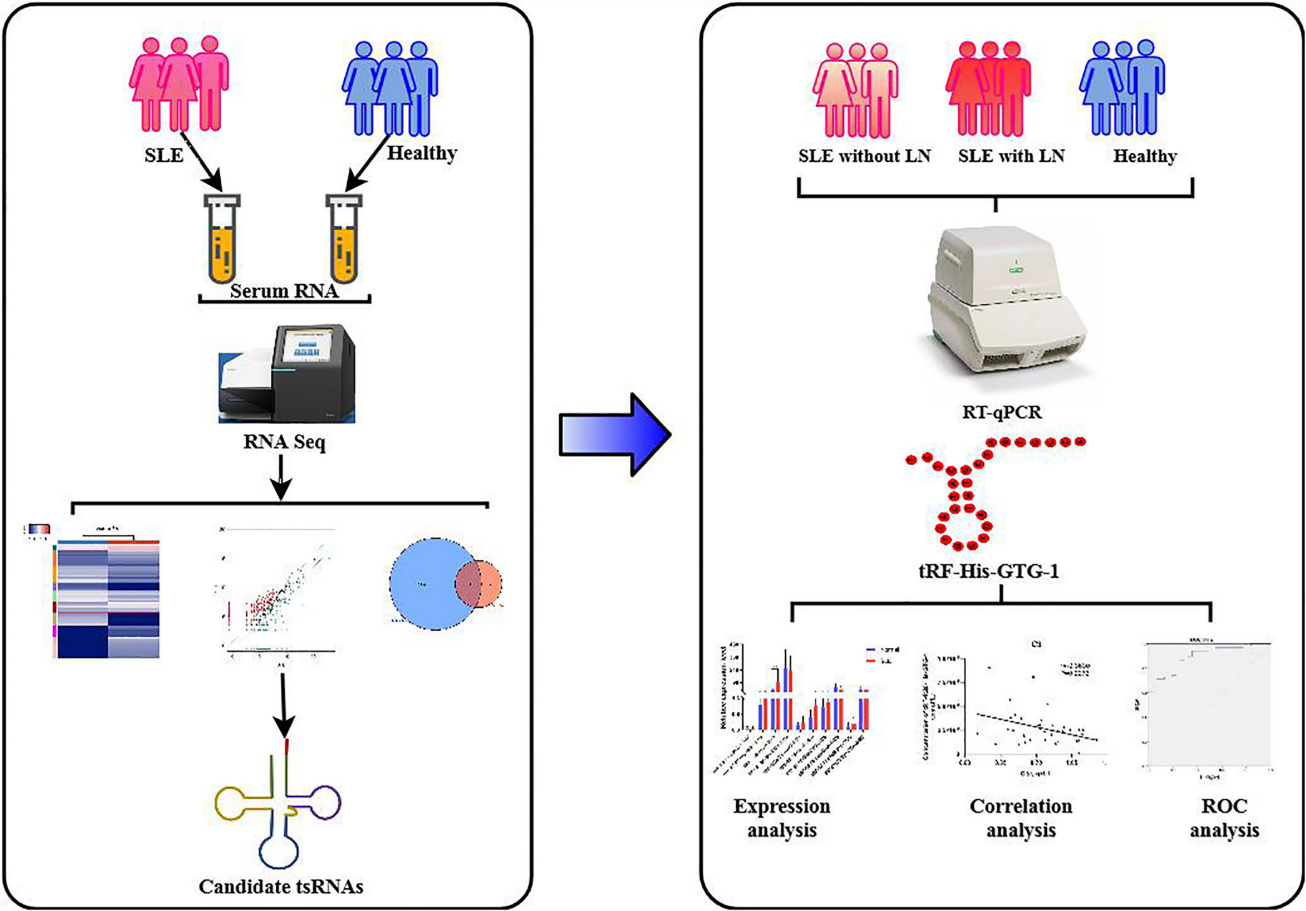
Workflow of the study.

This study was divided into two stages (**Figure 1**). In the training stage, to obtain an expression profile of serum tsRNAs specific for SLE, we employed a strategy that involved initial screening by small RNA (sRNA) sequencing and validation by qRT-PCR. At this stage, 24 SLE without LN patients, 33 SLE with LN patients, and 23 healthy controls were examined. Then, in the validation stage, selected tsRNAs were detected in a larger SLE cohort (52 SLE without LN patients, 83 SLE with LN patients, and 86 healthy controls).

### Patient Characteristics and Clinical Features

A total of 301 patients were divided into three groups; 76 patients with SLE without LN, 116 patients with SLE and LN, and 109 normal donors from Nanjing Drum Tower Hospital were enrolled in this study. The clinical characteristics of these patient samples are shown in **Table 1**. Patients involved in the study satisfied the revised criteria for SLE set up by the 1997 American College of Rheumatology (ACR) (27, 28). To record the SLE disease activity, we used the Systemic Lupus Erythematosus Disease Activity Index 2000 (SLEDAI) score. Ethics Committee of the Affiliated Drum Tower Hospital of Nanjing University Medical School approval was obtained for the study (ID: 2020-327-01). From every participant of the study, informed written consent was also obtained.

**Table 1:** Statistics of clinical information of testing group specimens.

	SLE without LN	SLE with LN	Normal
Female (percentage)	20 (83.33%)	28 (84.85%)	23 (100%)
Age (years), median (range)	37 (14–70)	35 (18–64)	34 (18–52)
ANuA(+) (percentage)	5 (29.41%)	10 (41.67%)	N/A
ASMA(+) (percentage)	7 (41.18%)	10 (41.67%)	N/A
AHA(+) (percentage)	6 (37.50%)	9 (37.50%)	N/A
ARPA(+) (percentage)	8 (47.06%)	8 (33.33%)	N/A
Urine protein (g/L), median (range)	0.16 (0.07–2.30)	2.61 (0.08–16.08)	N/A
C3 (g/L), median (range)	0.80 (0.32–1.29)	0.54 (0.13–1.41)	N/A
C4 (g/L), median (range)	0.16 (0.03–0.30)	0.08 (0.01–0.35)	N/A
IgG (g/L), median (range)	12.75 (7.50–112.00)	10.20 (4.10–28.80)	N/A
CRP (mg/L), median (range)	3.45 (0.20–52.90)	2.60 (0.40–56.70)	N/A
Anti-dsDNA (IU/L), median (range)	115.48 (0.001–922.60)	118.91 (0.001–1340.89)	N/A
Anti-β2-GP I (RU/ml), median (range)	7.00 (0.00–67.50)	4.20 (0.10–64.10)	N/A
WBC (10^9^/L), median (range)	5.80 (1.70–19.80)	6.10 (1.70–13.20)	N/A
RBC (10^12^/L), median (range)	3.73 (1.03–4.96)	2.93 (1.77–4.88)	N/A
HGB (g/L), median (range)	112 (34.00–153.00)	83.00 (54.00–151.00)	N/A
HCT (%), median (range)	33.60 (10.90–45.30)	25.30 (16.30–45.50)	N/A
PLT (10^9^/L), median (range)	181.00 (2.00–502.00)	139.00 (15.00–267.00)	N/A
Ne (10^9^/L), median (range)	3.55 (1.10–14.10)	3.90 (0.90–9.40)	N/A
Ly (10^9^/L), median (range)	1.10 (0.30–4.90)	0.80 (0.10–3.40)	N/A
Mo (10^9^/L), median (range)	0.40 (0.10–0.90)	0.30 (0.00–1.00)	N/A

ANuA, anti-nuclear antibody; ASMA, anti-Smith antibody; AHA; anti-histone antibody; ARPA, anti-ribosomal p protein antibody; C3, Complement C3; C4, Complement C4; IgG, immunoglobulin; CRP, C-reactive protein; WBC, white blood cell; RBC, red blood cell; HGB, hemoglobin; HCT, hematocrit; PLT, platelet; Ne, Neutrophils; Ly, lymphocyte; Mo, monocyte; N/A, not available.

### RNA Sequencing

#### Pretreatment of tsRNAs

The heavy decoration of tsRNAs by RNA modification is a main problem to deal with while constructing an sRNA sequence library. To construct a total RNA library, the following changes were made: 3’-aminoacyl was deacylated, 3’-cP (2’,3’-cyclic phosphate) was removed from 3’-OH to facilitate 3’ adaptor ligation, and 5’-OH (hydroxyl group) was phosphorylated to 5’-P for ligating a 5’-adaptor. m1A and m3C were demethylated to facilitate reverse transcription.

#### Library Preparation

The integrity of RNA samples was validated using agarose gel electrophoresis. Quantification of the RNA samples was achieved by using NanoDrop ND-1000 instrument. The removal of the RNA modifications, the main issue during the construction of the RNA sequence library, was carried out by adopting the pretreatment strategy as priorly explained. Agilent 2100 Bioanalyzer was used to quantify the completed libraries. Based on the quantification outcomes, the libraries were mixed in equal quantities for sequencing operation.

### Sequencing

For sequencing, the single-stranded DNAs were needed. To achieve that, 0.1 M NaOH solution was used to denature the DNA fragments in well-mixed libraries. The single-stranded DNA with 1.8 pM concentration was loaded onto the reagent cartridge. NextSeq system using NextSeq 500/550 V2 kit (#FC-404-2005, Illumina) was used for sequencing operation following the guidelines provided by the production company. The sequencing operation included 50 cycles.

### RT-qPCR

The serum samples were centrifuged at 3,500 rpm for 10 min before being collected and stored at -80°C until further analysis. High-quality RNA extraction from serum was achieved by TRIzol (Vazyme Biotech, Nanjing, China) method. The quantity and quality of total RNA were measured using OneDrop-2000 (NanoDrop Technologies). For the cDNA synthesis, 500 ng of total RNA was reverse transcribed using the miRNA 1st Strand cDNA Synthesis Kit (Vazyme Biotech). Ultrasensitive detection of tsRNAs was performed using the miRNA Universal SYBR qPCR Master Mix (Vazyme Biotech). The expression of each gene was determined, and the corresponding standard curve was drawn. Gene-specific primers (Genscript Biotech, Nanjing, China) are listed in **Supplementary Table S1**.

### Statistical Analyses

Statistical analyses were performed using an SPSS software, version 20.0 (SPSS Inc., Chicago, IL, USA), and GraphPad Prism software 7 (San Diego, CA, USA). Data are presented as the means ± SEMs, and statistical validations were achieved by harnessing t-test or one-way ANOVA followed by Bonferroni’s multiple comparisons test. P < 0.05 was nominated to represent the statistically significant differences.

### Data and Materials Availability

Raw data generated by tsRNA sequencing have been deposited to Gene Expression Omnibus (GEO) under the accession code GSE90524. The main findings of the study are included in the article and supplementary materials. Other data supporting the study findings can be acquired upon reasonable request from the corresponding authors.

## Results

### Ectopic Serum tsRNA Profiles in Systemic Lupus Erythematosus Patients

**Figure 2:**
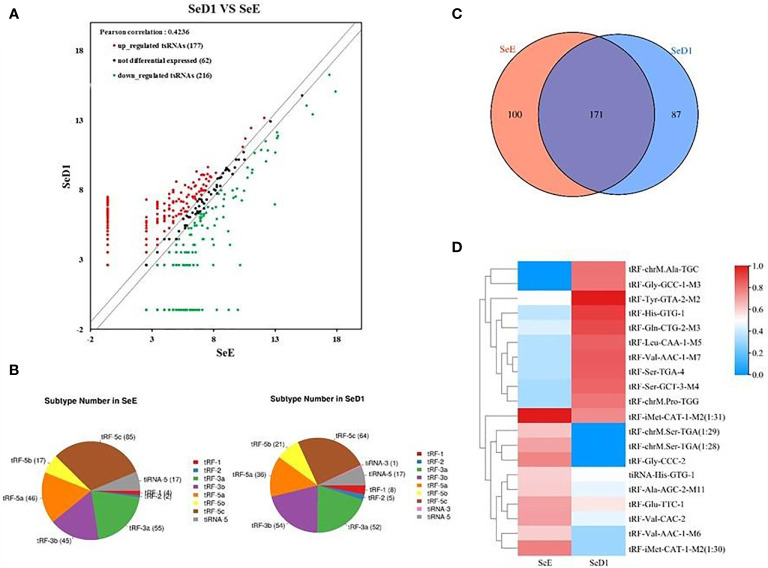
Analysis of differentially expressed tsRNAs in serum of systemic lupus erythematosus (SLE) patients. **(A)** Scatter plots of differentially expressed tsRNAs. tsRNAs above the top line (red dots, upregulation) or below the bottom line (green dots, downregulation) indicate more than 1.5-fold change between the two compared groups. Gray dots indicate non-differentially expressed tsRNAs. Statistical correlation was determined by Pearson correlation. **(B)** Distribution characteristics of tsRNA in SLE patients and healthy controls. **(C)** Venn distribution of tsRNAs in SLE patients and healthy controls. **(D)** Hierarchical clustering indicates the differences in tsRNA expression profiling between two groups.

The presence of stable tsRNAs in human serum has been confirmed by recent studies (26). Expression profile of serum tsRNAs can be potential fingerprints for various diseases (29–31). To identify the tsRNA profile in SLE patients, all participants were divided into three groups according to the clinical standard, including SLE without LN, SLE with LN, and healthy control. In this study, we employed a two-stage strategy to screen the SLE-specific tsRNAs (**Figure 1**). First, in the training stage, we started the search by comparing the tsRNA profiles in SLE serum with healthy controls. For tsRNA sequencing, 10 ml of serum pooled from 24 SLE patients and 23 healthy controls were extracted for RNA isolation, followed by tsRNA sequencing (GEO number: GSE90524). The basic characteristics of the patients are provided in **Table 1**. A total of 393 tsRNAs showed different expressions between SLE without LN groups and healthy controls in scatter plot analysis (**Figure 2A**). Distribution characteristics and Venn analyses of tsRNA also showed a difference in SLE without LN and healthy controls (**Figures 2B, C** **)**. tsRNAs that satisfied two conditions showed significant differential expression: CPM >100 in serum by sequencing detection and fold change >6 between two groups. As shown in **Table 2** and **Figure 2D**, the analysis resulted in 10 significant differentially expressed tsRNAs in serum from SLE patients compared with healthy controls.

**Table 2:** Sequencing information of 10 candidate tsRNAs.

tRF ID	tRF Sequence	tRF Length	Type	Fold Change	CPM
tRF-1:23-chrM.Ala-TGC	AAGGGCUUAGCUUAAUUAAAGUG	23	tRF-5b	272.28	212.32
tRF-27:42-Gly-GCC-1-M3	UCGCCUGCCACGCGGG	16	tRF-2	264.06	205.88
tRF-1:28-His-GTG-1	GCCGUGAUCGUAUAGUGGUUAGUACUCU	28	tRF-5c	12.25	456.80
tRF-68:85-Ser-GCT-3-M4	AUCCCAUCCUCGUCGCCA	18	tRF-3a	10.00	250.92
tRF-1:22-Val-AAC-1-M7	GUUUCCGUAGUGUAGUGGUUAU	22	tRF-5b	9.70	302.39
tRF-68:85-Ser-TGA-4	AUCCUGUCGGCUACGCCA	18	tRF-3a	9.28	289.52
tRF-57:75-Gln-CTG-2-M3	AGUCUCGGUGGAACCUCCA	19	tRF-3b	8.67	270.22
tRF-65:86-Leu-CAA-1-M5	UCGAAUCCCACUUCUGACACCA	22	tRF-3b	7.95	199.45
tRF-53:71-chrM.Pro-TGG	AAGACUUUUUCUCUGACCA	19	tRF-3b	6.57	765.63
tRF-57:76-Tyr-GTA-2-M2	GAUUCCGGCUCGAAGGACCA	20	tRF-3b	6.26	386.03

tsRNA, transfer RNA-derived small noncoding RNA; tRF,transfer RNA-derivrd fragment

### Identification of Differentially Expressed Serum tsRNAs in Systemic Lupus Erythematosus Patients

**Figure 3:**
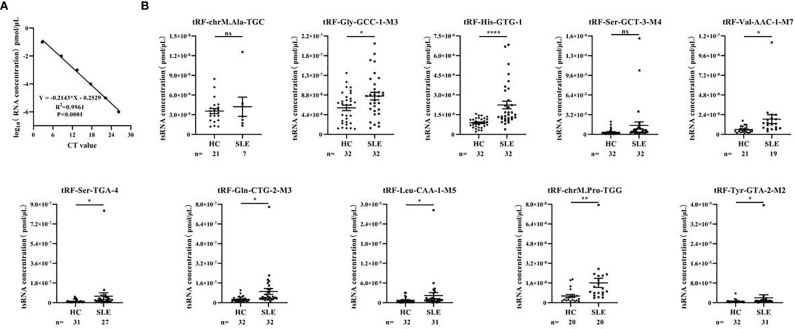
Identification of differentially expressed serum tsRNAs in systemic lupus erythematosus (SLE) patients. **(A)** Standard curve of tsRNA concentration. Statistical correlation was determined by linear regression and Pearson correlation. **(B)** RT-qPCR verification of 10 differentially expressed tsRNA in serum of SLE without lupus nephritis (LN) group and healthy control group. Statistical significance was determined by unpaired two-tailed t-test (*P < 0.05, **P < 0.01, ****P < 0.0001). ns, Non significant.

In the training stage, we then used RT-qPCR assay to validate the sequencing results. The absolute quantification was used to validate the expression of the top 10 upregulated tsRNAs using individual serum samples from 32 SLE without LN and 32 healthy controls by RT-qPCR (**Figure 3A**). RT-qPCR assay showed that tRF-His-GTG-1 was the most significantly elevated tsRNA in serum from SLE without LN compared to the control group (**Figure 3B**). In summary, tRF-His-GTG-1 was the candidate tsRNA that may be best classified between the SLE and control groups.

### Serum tRF-His-GTG-1 Is Specifically Upregulated in Systemic Lupus Erythematosus Patients

**Figure 4:**
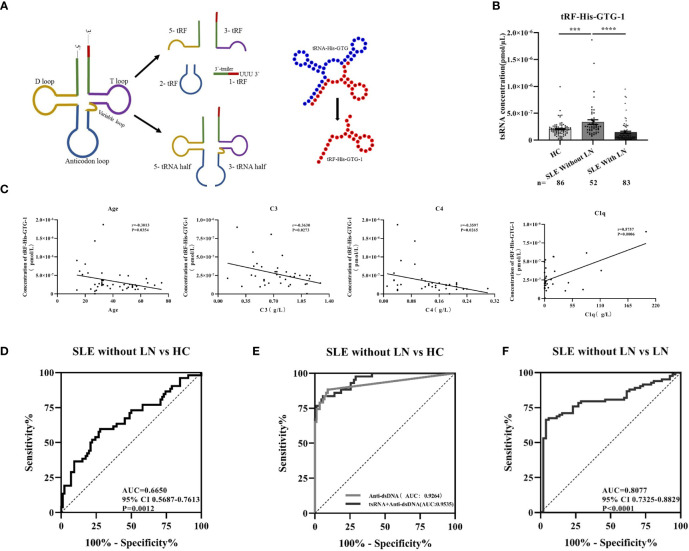
Diagnostic use of serum tRF-His-GTG-1 in systemic lupus erythematosus (SLE). **(A)** Schematic diagram of tRF-His-GTG-1 biogenesis and secondary structure. **(B)** RT-qPCR verification of tRF-His-GTG-1 in SLE without lupus nephritis (LN), SLE with LN, and normal. Statistical significance was determined by one-way ANOVA (***P < 0.001, ****P < 0.0001). **(C)** Analysis of the correlation between tRF-His-GTG-1 and age, C3, C4, and C1q. Statistical correlation was determined by linear regression and Pearson correlation. **(D)** Receiver operating characteristic (ROC) curve analysis of tRF-His-GTG-1 in SLE without LN and healthy controls. **(E)** ROC curve combined diagnostic analysis of tRF-His-GTG-1 and anti-dsDNA in SLE without LN and healthy controls. SPSS binary logistic regression was used to predict the probability of joint diagnosis. **(F)** ROC curve analysis of tRF-His-GTG-1 in SLE without LN group and SLE with LN group.

From tRNAs, two types of tsRNAs originate, tRNA-derived stress-induced RNAs (tiRNAs) and tRNA-derived fragments (tRFs). tiRNAs arise from the 5′ and 3′ tRNA halves consisting of about 30–40 nt. tRFs originate from mature and tRNA precursors by nucleases Dicer or angiogenin, etc. (10, 11), shorter than tiRNAs, and consist of 18–22 nt. Based on the position of sequence and the cutting site on tRNAs, four types of tRFs are recognized so far, including 5-tRFs, 3-tRFs, 1-tRFs, and 2-tRFs (18). In this study, the tRF-His-GTG-1 belongs to 5-tRFs (**Figure 4A**). To further test whether serum tRF-His-GTG-1 is upregulated and associated with progression in SLE patients, the level of tRF-His-GTG-1 was examined in large sample size, including 52 SLE without LN and 86 healthy controls (validation stage, **Table 3**). As shown in **Figure 4B**, the serum level of tRF-His-GTG-1 was significantly elevated in SLE without LN compared with healthy controls.

**Table 3:** Statistics of clinical information of validating group specimens.

	SLE without LN	SLE with LN	Normal
Female (percentage)	47 (90.38%)	74 (89.16%)	78 (90.70%)
Age (years), median (range)	38 (14–75)	45 (14–72)	33 (12–68)
SLEDAI-2K, median (range)	5 (0–13)	10 (0–19)	N/A
ANuA(+) (percentage)	14 (43.75%)	15 (32.61%)	N/A
ASMA(+) (percentage)	8 (25.00%)	15 (32.61%)	N/A
AHA(+) (percentage)	12 (37.50%)	21 (45.65%)	N/A
ARPA(+) (percentage)	9 (28.13%)	18 (39.13%)	N/A
Urine protein (g/L), median (range)	0.16 (0.01–8.59)	0.55 (0.05–11.18)	N/A
C3 (g/L), median (range)	0.75 (0.12–1.34)	0.78 (0.02–1.68)	N/A
C4 (g/L), median (range)	0.15 (0.01–0.30)	0.13 (0.01–0.42)	N/A
IgG (g/L), median (range)	13.50 (6.00–37.90)	12.50 (4.90–44.00)	N/A
CRP (mg/L), median (range)	4.25 (0.10–126.30)	4.50 (1.30–110.00)	N/A
Anti-dsDNA (IU/L), median (range)	157.61 (0.001–1465.91)	144.56 (0.001–1,105.95)	N/A
Anti-β2-GP I (RU/ml), median (range)	5.90 (0.20–271.90)	4.45 (0.10–57.10)	N/A
WBC (10^9^/L), median (range)	5.30 (0.50–36.30)	4.70 (0.50–20.50)	N/A
RBC (10^12^/L), median (range)	3.76 (1.01–38.00)	3.46 (1.51–5.91)	N/A
HGB (g/L), median (range)	110.00 (34.00–151.000	105.00 (55.00–162.00)	N/A
HCT (%), median (range)	33.30 (10.90–45.50)	31.70 (16.90–46.80)	N/A
PLT (10^9^/L), median (range)	196.00 (0.01–683.00)	150.00 (2.00–442.00)	N/A
Ne (10^9^/L), median (range)	3.40 (0.20–32.20)	3.30 (0.20–17.00)	N/A
Ly (10^9^/L), median (range)	1.10 (0.20–5.80)	0.90 (0.20–2.80)	N/A
Mo (10^9^/L), median (range)	0.40 (0.10–5.30)	0.30 (0.00–2.30)	N/A

ANuA, anti-nuclear antibody; ASMA, anti-Smith antibody; AHA; anti-histone antibody; ARPA, anti-ribosomal p protein antibody; C3,Complement C3;C4, Complement C4; IgG, immunoglobulin; CRP, C-reactive protein; WBC, white blood cell; RBC, red blood cell; HGB, hemoglobin; HCT, hematocrit; PLT, platelet; Ne, Neutrophils; Ly, lymphocyte; Mo, monocyte; N/A, not available.

### Diagnostic Use of Serum tRF-His-GTG-1 in Systemic Lupus Erythematosus

To verify the value of tRF-His-GTG-1 for clinical practice, we used ROC curve analysis and assessed the sensitivity and specificity of prediction based on the risk scores. As shown in **Figure 4D**, serum tRF-His-GTG-1 in SLE without LN had higher diagnostic use with area under the curve (AUC) of 0.67 (95% CI, 0.57–0.76) and performance (sensitivity 59.62%, specificity 72.09%) relative to the healthy group. To further demonstrate the clinical significance of tRF-His-GTG-1 expression in SLE patients, we investigated the association between the tsRNA level and various clinical indicators in a total of 52 SLE serum samples. The results showed that high serum tRF-His-GTG-1 was negatively associated with age and complement C3 and C4 but positively correlated with C1q (**Figure 4C**). Moreover, tRF-His-GTG-1 had no association with SLEDAI, anti-dsDNA, anti-β2, C3b, immunoglobulin G (IgG), C-reactive protein (CRP), proteinuria (**Supplementary Figure S2A**). Then, we explore the predictive accuracy of the tRF-His-GTG-1 combined with clinical indicators. ROC curve was used to analyze the diagnostic efficacy of tRF-His-GTG-1 combined with anti-dsDNA antibody C1q, C3b, proteinuria (**Figure 4D** and **Supplementary Figure S2B**). In the combined analysis, a logistic regression model with tRF-His-GTG-1 and anti-dsDNA antibody resulted in a higher AUC of 0.95 (95% CI = 0.92–0.99) and higher performance (sensitivity 83.72%, specificity 94.19%) compared with a single anti-dsDNA antibody (**Figure 4E**), highlighting the diagnostic performance of the combination of tRF-His-GTG-1 and anti-dsDNA antibody for identifying SLE.

### Serum tRF-His-GTG-1 as Biomarkers to Distinguish Lupus Nephritis From Systemic Lupus Erythematosus

To further explore whether serum tRF-His-GTG-1 level is related to the progression of SLE, we then performed qRT-PCR to assay serum samples from 83 SLE with LN patients. The expression of the tRF-His-GTG-1 in serum was remarkably decreased in SLE with LN than in SLE patients without LN (**Figure 4B**). ROC curves were performed to determine the diagnostic characteristics of serum tRF-His-GTG-1. Surprisingly, serum tRF-His-GTG-1 in SLE with LN group showed a higher diagnostic value with AUC of 0.81 (95% CI, 0.73–0.88) and performance (sensitivity 66.27%, specificity 96.15%) relative to SLE without LN group (**Figure 4F**). These results demonstrate that the levels of tRF-His-GTG-1 can serve as a promising diagnostic indicator to distinguish the LN patients among all SLE patients.

### tRF-His-GTG-1 Function in Systemic Lupus Erythematosus

**Figure 5:**
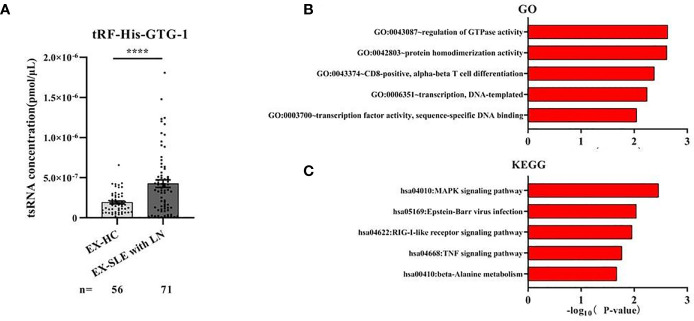
The biological functions of the tRF-His-GTG-1. **(A)** Expression analysis of tRF-His-GTG-1 in exosomes of healthy group and systemic lupus erythematosus (SLE) group. Statistical significance was determined by unpaired two-tailed t-test (****P < 0.0001). **(B)** Gene Ontology (GO) term analysis of tRF-His-GTG-1. **(C)** Kyoto Encyclopedia of Genes and Genomes (KEGG) signal pathway analysis of tRF-His-GTG-1.

The existing forms of tRF-His-GTG-1 in serum were analyzed to explore their potential functions. Intensive studies suggested that exosomes carry small noncoding RNAs that can be delivered into recipient cells where they function as endogenous RNAs, simultaneously regulating multiple target genes or signaling events (32). We first determined whether serum tRF-His-GTG-1 was circulating *via* exosomes. We isolated exosomes from SLE patient serum. Our data suggested that tRF-His-GTG-1 was high in serum exosomes (**Figure 5A**). To investigate the potential functions of tRF-His-GTG-1, we then used target prediction tools, RNAhybrid and Targetscan, to analyze its target genes. Then, Gene Ontology (GO) and Kyoto Encyclopedia of Genes and Genomes (KEGG) analysis were performed to identify biological processes associated with the tsRNA target genes [P < 0.001, false discovery rate (FDR) <0.05]. The high-enrichment GO terms targeted by tRF-His-GTG-1 include regulation of GTPase activity, protein homodimerization activity, and alpha-beta T-cell differentiation (**Figure 5B**). KEGG annotation showed that mitogen-activated protein kinase (MAPK) signaling pathway, Epstein–Barr virus infection, RIG-like receptor signaling pathway, and tumor necrosis factor (TNF) signaling pathway were enriched (**Figure 5C**). Most of the GO items and signaling pathways were associated with immunoregulation. These bioinformatics findings may add to the evidence that the tRF-His-GTG-1 can modulate immunity in SLE patients.

## Discussion

SLE is an autoimmune disease that affects multiple organs and tissues. Its clinical manifestations are abnormal activation of lymphocytes (33, 34) and abnormal buildup of autoantibodies (35). Kidney is one of the most crucial organs in the human body, and LN has a huge negative impact on the kidney. Within 5 years of diagnosis, 10% of individuals will develop ESRD. The poor survival rate of the diseases is a huge obstacle for current treatment strategies (8, 36, 37). Early detection and prediction of progression are both rapidly growing areas in SLE research to improve therapeutic efficacy. Currently, the clinical indicators of SLE diagnosis or progression monitoring, such as anti-dsDNA antibody, urinary protein, serum creatinine clearance rate, complements C3 and C4, or renal biopsy (38–40), have obvious limitations and have failed to fulfill clinical needs. Therefore, new noninvasive biomarkers with higher sensitivity and specificity for the early detection of SLE are highly desired.

Liquid biopsies for detecting novel biomarkers in circulation provide an attractive alternative for the diagnosis of diseases. Recently, circulating miRNAs and DNAs have been comprehensively studied as biomarkers for a variety of diseases (21, 22, 41). The discovery of short noncoding RNAs was one of the most major advances in recent decades (42, 43). Among them, tsRNAs, a novel type of small noncoding RNA generated by the cleavage of tRNA or pre-tRNA, have drawn great attention. Although dysregulation of tsRNAs had been observed in a variety of cancers (12–16), neurodegenerative diseases (17, 18), and metabolic diseases (19, 20), the profiling and role of tsRNAs in autoimmune diseases have not yet been studied. tsRNAs had also been found to be stable in the circulation and are likely to be promising disease biomarkers for two reasons: (1) tsRNAs harbor various RNA modifications (inherited from their tRNA precursors) than other small noncoding RNAs in circulation (11, 44), which may improve RNA stability and provide more information; (2) tsRNA signatures may sensitively change under variable stress conditions (45), the environment to which SLE patients are commonly exposed. This leads us to suspect that the tsRNA signatures may be more sensitive to reflect the progression of SLE disease. We adopted a “proof-of-principle” approach in this study. Particular SLE-specific serum tsRNAs were identified individually through high-throughput RNA sequencing, validated by using qRT-PCR validation sets at the individual level and then analyzed in combination with other clinical markers. In light of the abovementioned approach, we identified that tRF-His-GTG-1 combined with anti-dsDNA had high sensitivity and specificity in distinguishing SLE patients from healthy controls. These combined markers exhibited 83.72% sensitivity and 94.19% specificity. We anticipate that if these combined serum markers are validated by future studies, with large patient population size, it can advance in randomized clinical trials and can be harnessed for efficient and early detection of SLE.

For efficient diagnosis of LN and LN subtype identification, the current gold standard is kidney biopsy (8). However, the procedure is invasive, risky, and not easy to be repeated. The 24-h urine protein quantification is used frequently but had many drawbacks, such as inaccurate timing, partial urine sample loss during urine retention, and poor patient compliance for urine protein tests. It is very difficult to detect the occurrence of LN in clinical SLE patients. Hence, another unique extension of our study is that serum tRF-His-GTG-1 could serve as a noninvasive biomarker for distinguishing LN in SLE patients. The serum tRF-His-GTG-1 signature had an excellent ability to distinguish LN patients from SLE patients with high sensitivity (66.27%) and specificity (96.15%), better than proteinuria (sensitivity 58.21%, specificity 100%). These results highlight that noninvasive serum tRF-His-GTG-1 could be used to distinguish SLE with LN or SLE without LN. Although the mechanism of decreased serum tRF-His-GTG-1 in SLE with LN has not been identified, we guess that damaged kidneys may leak more tRF-His-GTG-1 into urine. Besides, the accumulation of renal disease status may also reshape the immune microenvironment *in vivo*, and it will significantly influence the expression profile of serum tsRNAs. It is therefore recommended that future studies in multicenter clinical trials and molecular mechanism should be adopted to carefully assess the clinical utility of serum tsRNAs as diagnostic biomarkers.

In summary, we have introduced a serum tsRNA signature in SLE patients for the first time. In particular, we have demonstrated that combined serum tRF-His-GTG-1 and anti-dsDNA antibodies can serve as noninvasive biomarkers for diagnosing SLE. More importantly, the profile of tRF-His-GTG-1 may also function as a warning marker for LN in SLE patients. These results can provide an impetus for future studies to further explore serum tsRNAs for potential clinical applications and to understand their biological functions in depth.

## Data Availability Statement

The original contributions presented in the study are publicly available. These data can be found here: https://www.ncbi.nlm.nih.gov/geo/GSE179950.

## Author Contributions

PY, XZ, and SC contributed equally to this work. YW is the chief designer of the whole experiment. All authors contributed to the article and approved the submitted version.

## Funding

This work was supported by the National Natural Science Foundation of China (No. 32000549), Training Program of the Major Research Plan of the National Natural Science Foundation of China (No. 92049109), National Natural Science Foundation of China (Nos. 32000549 and 61971216), Key Research and Development Project of Jiangsu Province (Nos. BE2019603 and BE2020768), High-level Health Talent Project of Jiangsu Province (No. LGY2019001).

## Conflict of Interest

The authors declare that the research was conducted in the absence of any commercial or financial relationships that could be construed as a potential conflict of interest.

## Publisher’s Note

All claims expressed in this article are solely those of the authors and do not necessarily represent those of their affiliated organizations, or those of the publisher, the editors and the reviewers. Any product that may be evaluated in this article, or claim that may be made by its manufacturer, is not guaranteed or endorsed by the publisher.
