# Supplementary figures and images for: A Novel Serum tsRNA for Diagnosis and Prediction of Nephritis in SLE
Source: Front Immunol. 2021 Nov 11;12:735105. doi: 10.3389/fimmu.2021.735105 (PMC8632637; doi:10.3389/fimmu.2021.735105)

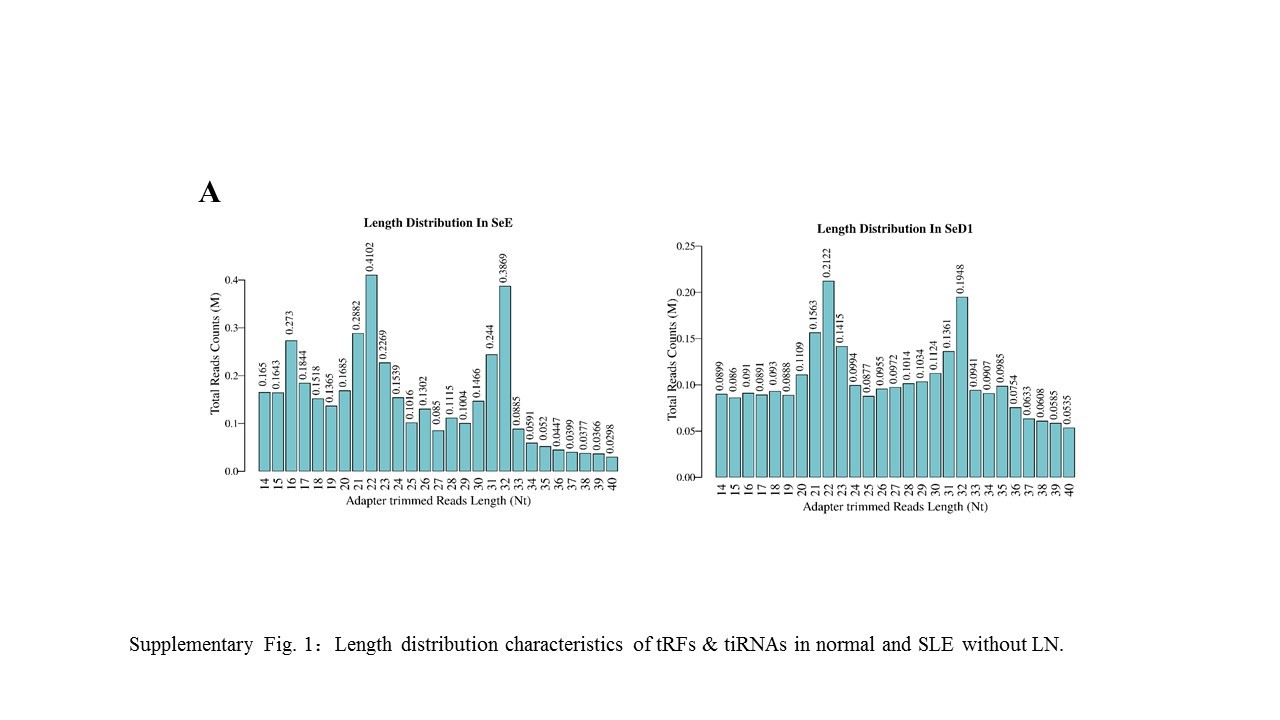

Supplement: Supplementary file 1 [file Image_1.jpeg]

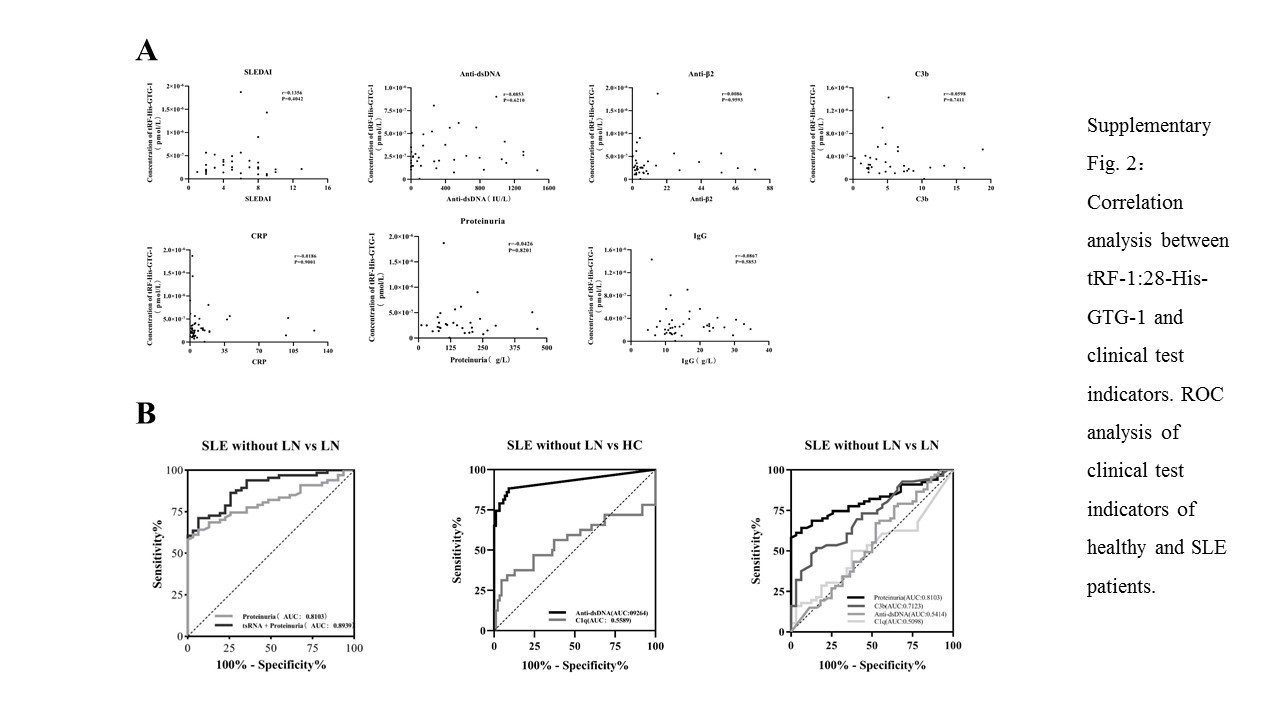

Supplement: Supplementary file 2 [file Image_2.jpeg]
