# Supplementary material for: A Novel Serum tsRNA for Diagnosis and Prediction of Nephritis in SLE
Source: Front Immunol. 2021 Nov 11;12:735105. doi: 10.3389/fimmu.2021.735105 (PMC8632637; doi:10.3389/fimmu.2021.735105)
Supplement: Supplementary file 3 [file Table_1.docx]

Supplementary Table 1: Specific primer information for 10 candidate tsRNAs.

Supplementary Table 2: Characteristic information of receiver operating curve(ROC). ROC P value from paired two-tailed t-test.
